# Low-grade Myofibroblastic sarcoma: clinical and imaging findings

**DOI:** 10.1186/s12880-018-0287-z

**Published:** 2019-05-02

**Authors:** Lu Wang, Ling-Xia Li, De-Qiang Chen, Lin Yang, Shu-Kui Li, Cai Cheng

**Affiliations:** 1 Department of Orthopedic, Cang Zhou central Hospital, Cang Zhou, 061014 China; 2 Department of Clinical pharmacology, Cang Zhou People’s Hospital, Cang Zhou, 061000 China; 3 Department of Radiology, Cang Zhou central Hospital, Cang Zhou, 061014 China

**Keywords:** Low-grade myofibroblastic sarcoma (LGMS), Bones, Soft tissues, CT, MRI

## Abstract

**Background:**

Low-grade myofibroblastic sarcoma (LGMS) is a rare type of tumor. Previous research has paid much attention to reporting pathological analyses of LGMS. However, only few systematic clinical and/or radiological studies have been conducted.

**Methods:**

This study recruited 14 cases (8 males and 6 females) of LGMS. X-ray or computer tomography (CT) scan were performed on 11 cases. MRI was performed on 5 cases.

**Results:**

X-Ray and CT scan: Five cases developed LGMS in bones, including 3 cases in the distal femur, 1 in the right shoulder blade, and another 1 in the right inferior ramus. Massive infiltrative and vermiform bone destruction with poorly-circumscribed lesion margins and partial soft tissue masses were observed. The other 9 cases were developed in soft tissues. Out of them, 4 cases presented slightly irregular hyper- or lower-density masses with poorly-circumscribed margins. 2 cases presented massive calcification and ossification. Significant enhancement was observed in 1 case, while no obvious enhancement was seen in the other 2 cases.

MRI: MR images of 5 cases revealed homogeneous iso- or hyper-signal intensity on T1WI and homogeneous or heterogeneous hyper-signal intensity on T2WI. Enhanced MRI revealed homogeneous enhancement in 2 cases and rim enhancement in 1 case.

**Conclusions:**

Our findings show that LGMS is characterized by invasiveness, metastases and calcification. Different radiological tools should be employed to make an accurate diagnosis.

## Background

Low-grade myofibroblastic sarcoma (LGMS) is a rare type of malignant myofibroblastic tumor. Though it may occur to any body part, the most common location is the limbs, head and neck region, particularly the tongue and mouth [1]. Previously published literature mainly focused on reporting pathological analyses of LGMS, while only few systematic clinical and/or radiological studies of this disease have been conducted.

This study recruited 14 cases of LGMS from two hospitals (one of the 14 participants had converted to LGMS from multiple relapses of an inflammatory myofibroblastic tumor). The imaging findings and biological characteristics were different from those of previous studies. Therefore, we report them in this article, hoping to provide further insights into LGMS.

## Methods

### Image acquisition

Esophageal angiography was performed on the participant with LGMS in the pyriform sinus (Case 4). X-ray or computer tomography (CT) scan were performed on 11 cases before surgery. Dual energy CT (Siemens SOMATOM Definition) images revealed slice thickness of 5–10 mm, tube voltage of 120 kV, tube current of 559 mA, pitch of 3.2, and gantry perpendicular to the CT table. Multi-planar reformatting of CT images was performed by a workstation (Advantage Workstation 4.3; GE Healthcare, Waukesha, WI, USA).

Magnetic resonance imaging (MRI), without and with contrast materials, was performed on five cases using a 3.0 T MRI scanner (GE Signa Excite). Dedicated coils were used to image different body parts and the regions of interest. MR images were acquired with spin-echo pulse sequences. Axial view: T_1_WI:TR/TE 440/8.2 ms, T_2_WI: TR/TE: 4000/142.5 ms; slice thickness: 5 mm; NEX:4.0, FOV; 38 cm × 38 cm; and matrix: 256 ×224~ 512 × 446. Fat-suppressed T1-weighted transverse images: a gadopentetate dimeglumine of 0.1 mmol/kg (Magnevist, Schering, Berlin, Germany) was injected intravenously into each patient with an injection rate of 2.0 ml/s. Axial view: T_1_WI: TR/TE 560/8.0 ms; slice thickness:6 mm; FOV 38 cm×38 cm; and matrix: 320×192~ 512×446. Corona view: T_1_WI: TR/TE 560/8.2 ms; slice thickness:5 mm; FOV 40 cm×40 cm; and matrix: 320×192~ 512×446.

### Image analysis

Analysis of all images was done by two board-certified radiologists specializing in musculoskeletal imaging. CT images were analyzed on tumor location, morphology, size, margins, density and the presence of calcification. MR images were evaluated for tumor morphology, margins, signal intensity and enhancement, necrosis, hemorrhage, and peritumoral edema. All imaging findings were in line with those of pathological analysis.

### Pathological analysis

Tumor specimens obtained after surgical excision were fixed in a 10% formaldehyde solution for 24 h for dehydration, and the paraffin-embedded specimens were sliced and stained with hematoxylin and eosin (H&E). Immunohistochemistry streptavidin-biotin staining (S-P) link staining with 3,3′-Diaminobenzidine (DAB) color rendering was performed on some specimens. Cytoplasmic brown precipitation was considered to be positive. Histopathological characteristics were analyzed by 2 board-certified pathologists specializing in musculoskeletal specialty.

## Results

### Clinical data

A total of 14 LGMS cases were recruited from two hospitals from April 2005 to July 2015, including the hospital where we worked. These 14 participants included 8 male and 6 female patients with a history of from 1 to 8 years. All of them had been clinically diagnosed and undergone surgical procedures, pathological analysis, and post-operation follow-ups in the two hospitals. Details of tumor location and medical history are listed in Table 1. The tumors located in bones (5 cases) were poorly circumscribed, therefore, the diameter of the lesions could not be accurately measured. The maximum diameter of the tumor lesions in soft tissues (the other 9 cases) ranged from 4.3 to 13.5 cm, with an average maximum diameter of 7.8 cm. Three out of the 14 cases (21.4%) suffered from local recurrence within 7 months to 4 years after surgical resection (Case 3, 10 and 14). One of these 3 recurrent cases (Case 14) suffered 8 recurrences within 8 years. The participant was initially diagnosed with inflammatory myofibroblastic tumor based on pathological examinations after the first to third surgeries, but was eventually diagnosed with LGMS based on the pathological analyses after the fourth to eighth surgeries. Bone metastasis recurred to another participant (Case 10) twice within 17 months after the second surgery. In addition, distant metastasis, primarily in the lungs and bones, occurred in 5 out of 14 cases (35.7%), either before surgeries or within 11 to 56 months after surgical excision.

**Table 1:** The Clinical and Imaging manifestations of LGMS

No	location	Medical history	size(cm)	X-Ray/CT	CT value (Pre- contrast)	CT value (Post- contrast)	T1WI	T2WI	Internal mass enhancementa	Follow-up
1	Right scapula	6 months	13.0 × 13.5	Irregular mass with a large of calcium and ossification	22–31	31–39	/	/	/	pulmonary meta-stasis befor operation
2	Left distal femur	8 months	–	osteolytic da-mage with cortex destruction	/	/	/	/	/	pulmonary meta-stasis after 8 months of operation
3	Right shoulder	1 year	8.9 × 6.8	/	/	/	Iso signal	High signal	Homogenous	Recurrence after 4 years of operation
4	Laryngo -pharynx	3 months	3.9 × 4.4	filling defect	/	/	/	/	/	/
5	Left armpit	17 months	4.3 × 4.0	Irregular mass	61	77	Iso signal	Heterogene-ous high signal with dark internal septation	Homogenous	/
6	Left breast	1 year	6.2 × 3.5	High density irregular mass	/	/	/	/	/	bone metastasis after 11 months of operation
7	Right distal femur	4 months	–	osteolytic da-mage with cortex destruction	/	/	Iso signal	Heterogene-ous high signal	Rim enhancement	pulmonary meta-stasis after 56 months of operation
8	Left thigh	6 months	6.6 × 4.4	Irregular mass with a large of calcium and ossification	44	92	/	/	/	/
9	Right partesiliaca	2 years	–	ossificationabove the ri-ght greater trochanter of femur	/	/	/	/	/	/
10	Left distal femur	2 years	–	osteolytic dam-age with cortex destruction	/	/	/	/	/	Reccurence and bone metastasis after 17 months of operation
11	Right remi inferior ossis pubis	10 years	–	expansive da-mage with cortex destruction	/	/	/	/	/	/
12	left lobe of liver	4 days	11.2 × 8.1	Low density lobulated mass	28	39	/	/	/	/
13	Partes oralis	6 months	–	/	/	/	Slight high signal	Heterogene-ous high signal	Rim enhancement	/
14	Right thigh	Recur 8 times in 8 years	5.6 × 7.4	/	/	/	Slight low signal	High signal with dark internal septation	Heterogeneous	IMT translate into LGMS after reccurence for 3 times

-:cannot be measured

*IMT* inflammatory myofibroblastic tumor

### X-ray and CT scan findings

X-ray and CT scan results are illustrated in Table 1. Eleven out of the 14 cases (78.6%) had pre-operational X-ray or CT scan, and 4 of the 11 received contrast-enhanced CT scan.

**Fig. 1:**
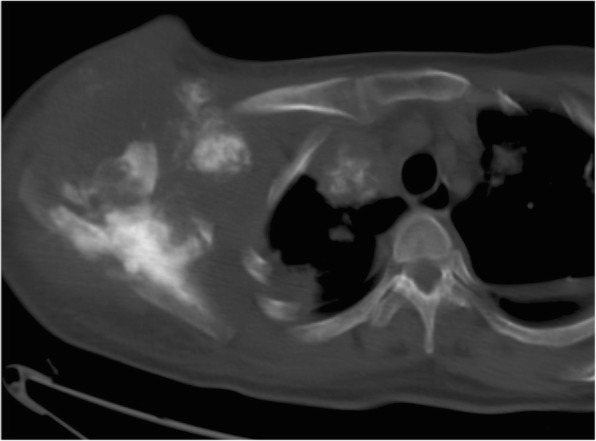
LGMS of the left breast. Mammography revealed an irregular high-density mass without calcification. No thickening and/or increase was observed in the surrounding breast trabeculae. and no swelling in the axillary lymph nodes

**Fig. 2:**
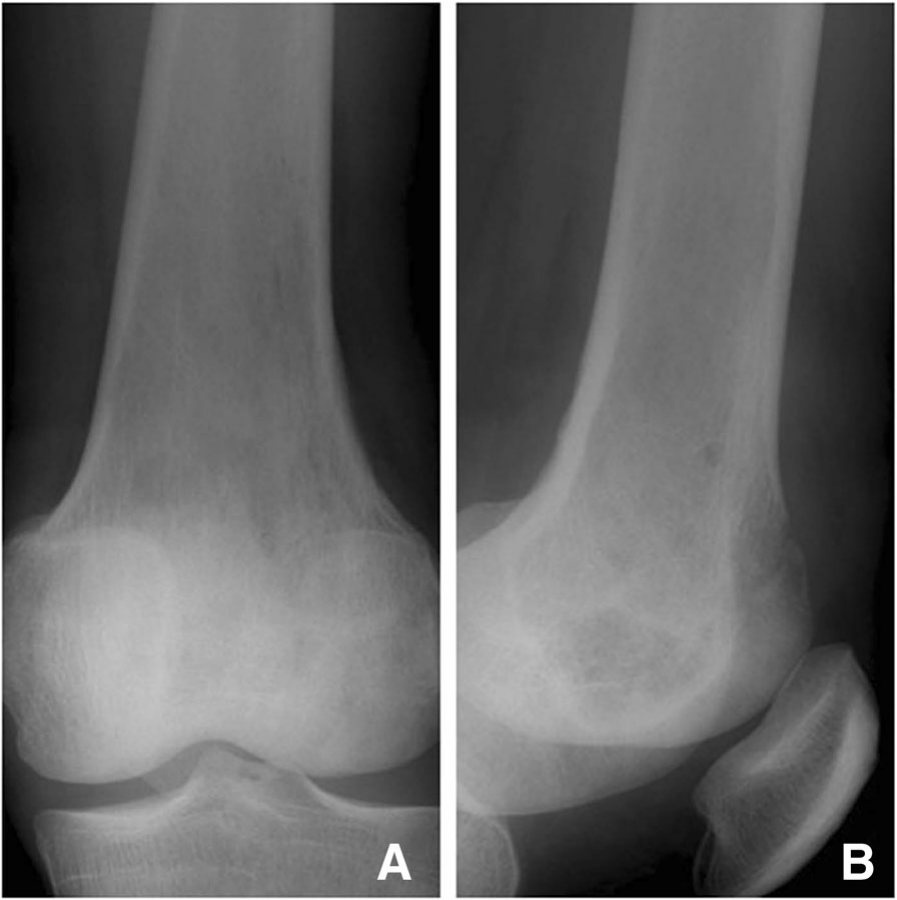
LGMS of the left distal femur. X-ray revealed osteolytic damage and cortex destruction of the left distal femur (**a, b**)

**Fig. 3:**
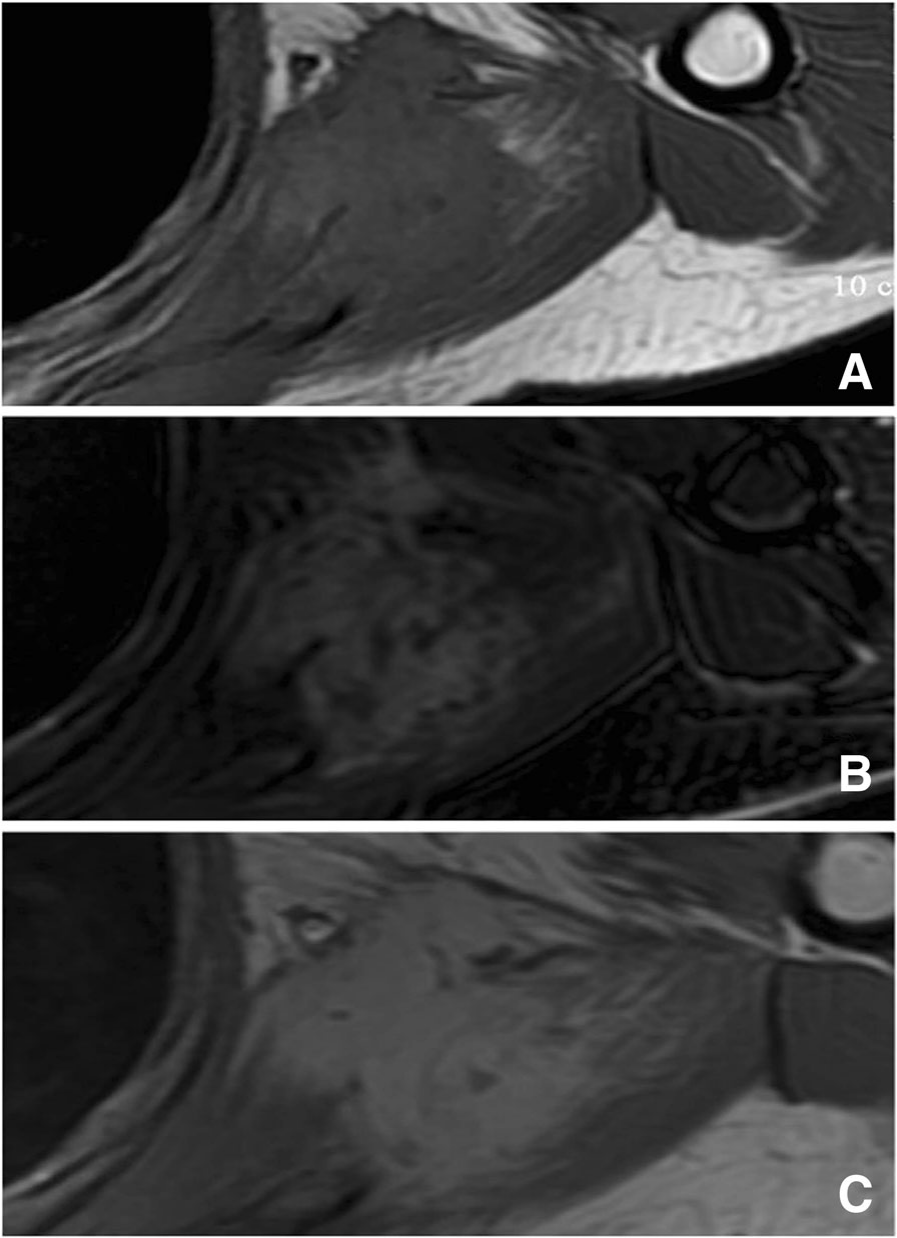
LGMS of the left lobe of the liver. CT scan revealed a low-density mass (**a**). The contrast-enhanced images showed heterogeneous enhancement at the early period (**b**). Delayed contrast-enhanced images revealed partially-filled concentric enhancement

**Fig. 4:**
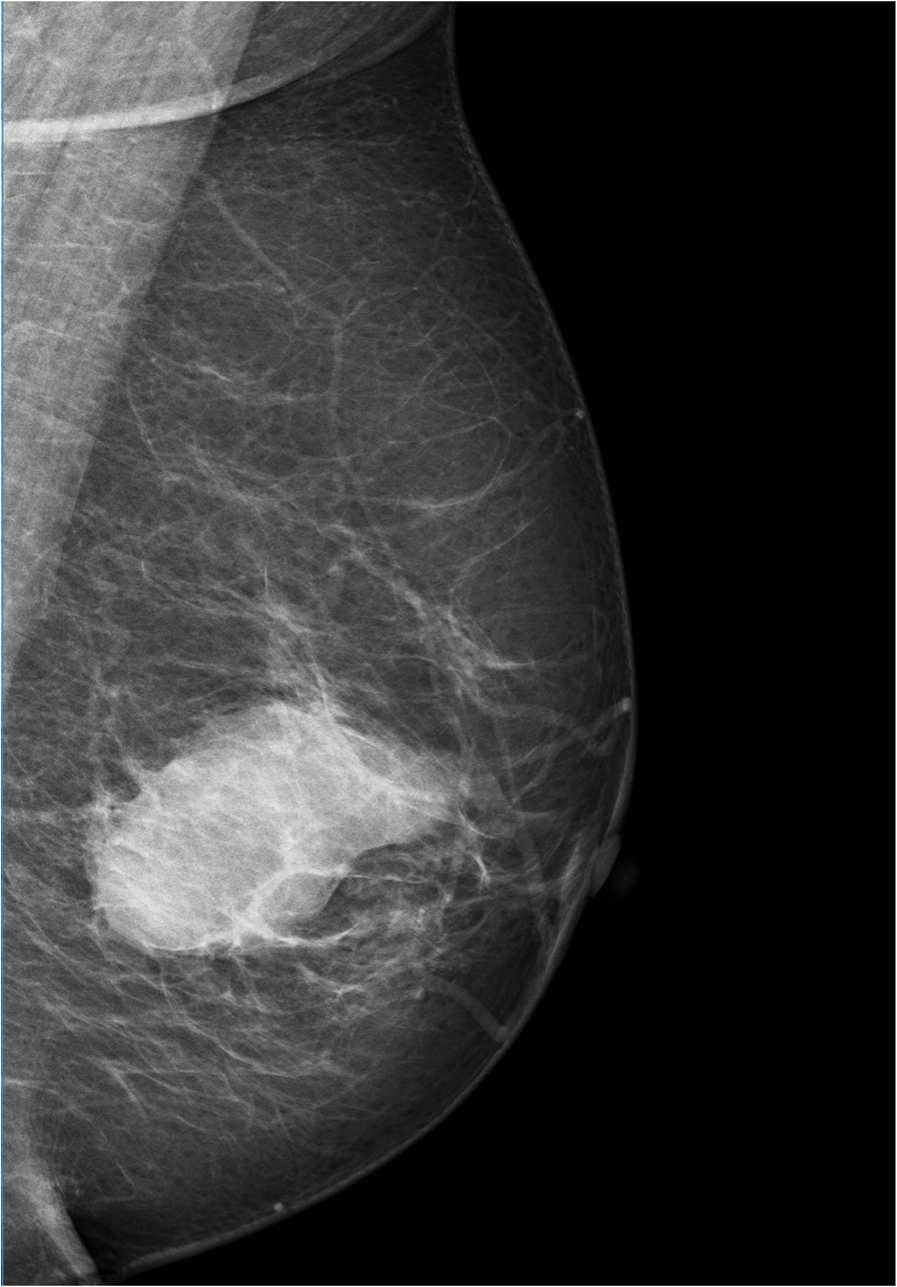
X-ray revealed osteolytic damage and cortex destruction of the left distal femur (**a**). T2-weighted MRI revealed a heterogeneous high signal mass (**b**). Gadolinium-enhanced T1-weighted fat-suppression images revealed rim enhancement (**c**)

LGMS was located in bones in five cases, including 3 in the distal femur, 1 in the right shoulder blade, and 1 in the right inferior ramus of the pubis symphysis (Fig. 1). Scan results of tumors in bones revealed massive infiltrative or vermiform bone destruction with poorly distinguishable lesion margins (Figs. 2, and 3), erosion of bone cortex, and partial soft tissue masses. Scan of the tumor in the right shoulder blade revealed osteolytic bone destruction with obvious bone sclerosis in adjacent bones and a large amount of ossification in neighbor soft tissue masses (Fig.4). Multiple metastases were observed in both lungs in this patient, and some of the metastases were with severe ossification (Fig.4). However, no obvious enhancement of the right shoulder blade tumor was seen in the contrast-enhanced CT scan image (enhanced CT number: < 10 HU). Scan of the tumors in the distal femur revealed multi-cystic bone destruction with significant swelling, boarder erosion and damage, and concurrent soft tissue masses.

**Fig. 5:**
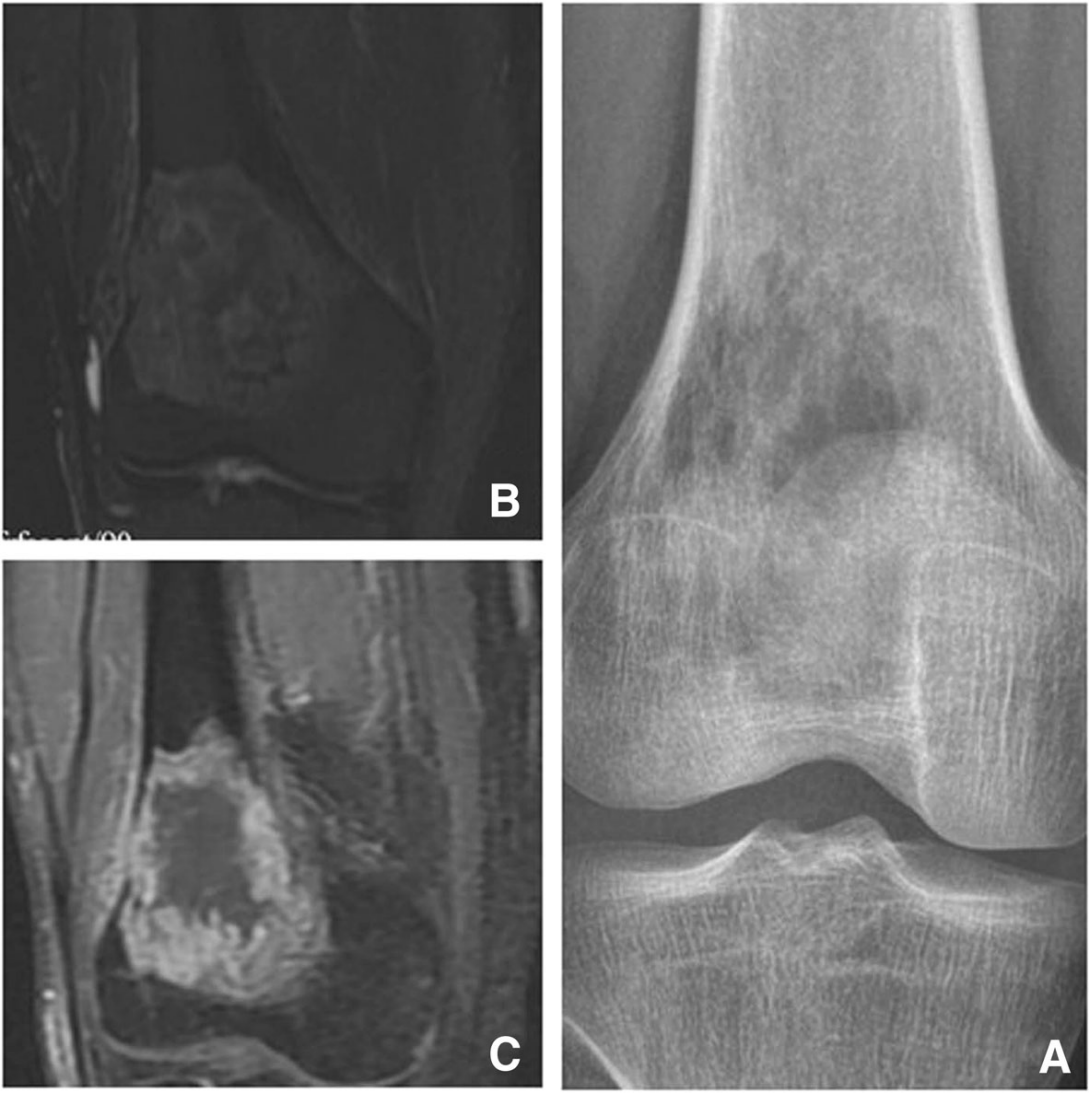
LGMS of the right shoulder. An irregular soft tissue mass in right shoulder with equal T1 and long T2 was observed (**a**, **b**). Enhanced MRI revealed a homogenous mass (**c**)

**Fig. 6:**
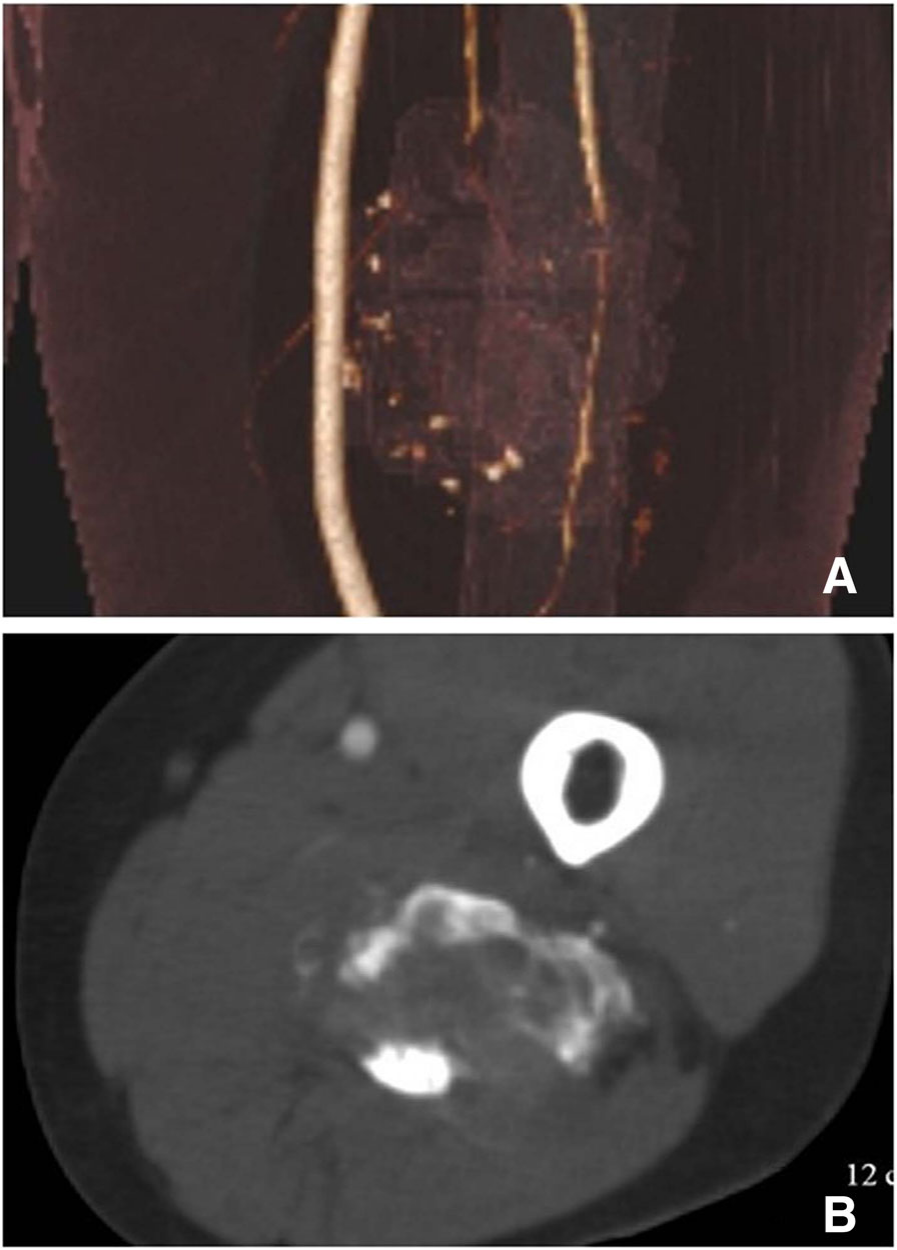
LGMS of the left thigh. CT scan revealed an irregular mass (**a**) with calcification inside (**b**)

**Fig. 7:**
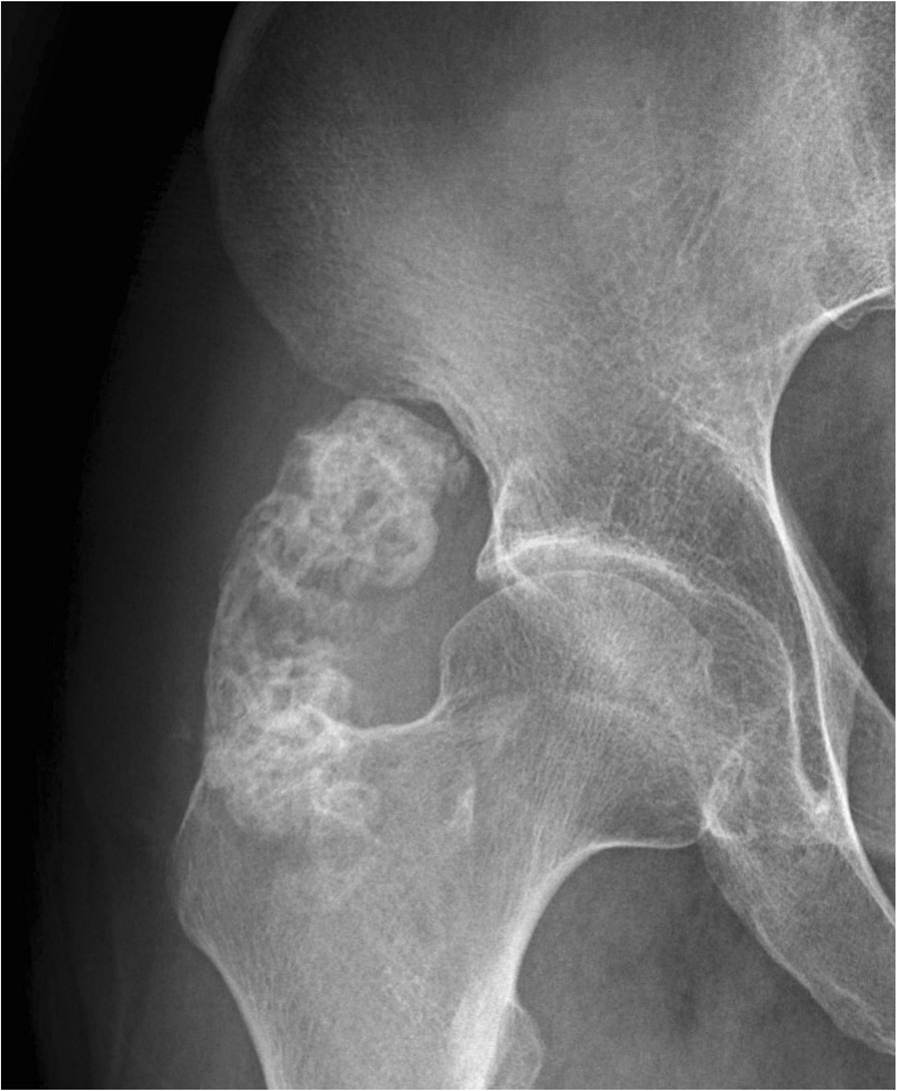
X-ray revealed extensive damage and cortex destruction of the right remi inferior ossis pubis

**Fig. 8:**
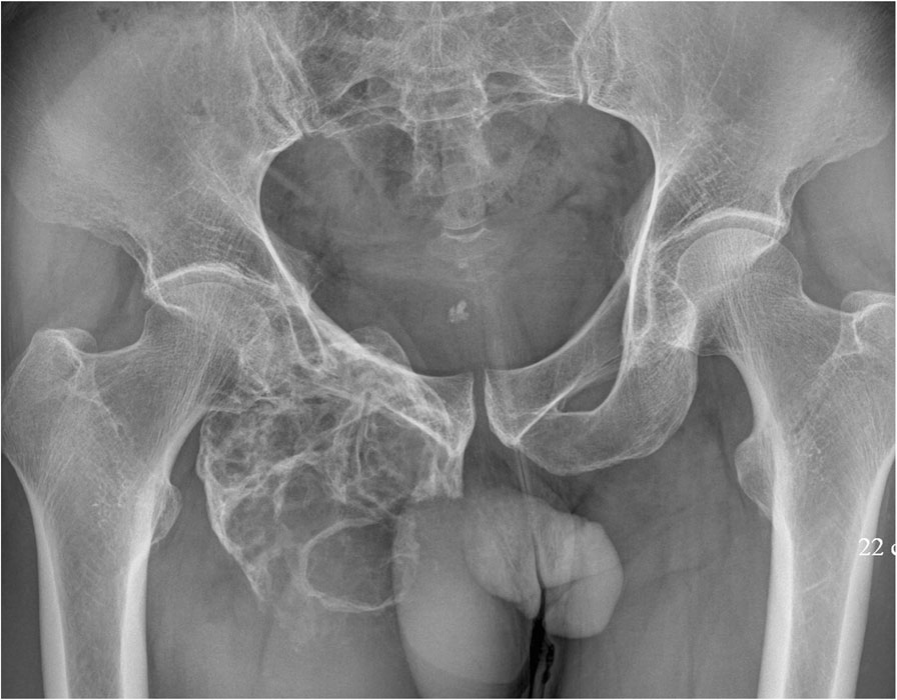
LGMS of the right shoulder blade. Pre-operational CT scan revealed an irregular mass with massive calcification and ossification, and pulmonary metastasis

There were a total of 9 cases of LGMS in soft tissues. Preoperational esophagus angiography performed on Case 4 revealed lobulated filling defects with smooth margins in the right pyriform sinus and epiglottic vallecula region. Preoperational X-ray was performed on Case 6 who had a lobulated mass with well-circumscribed margins in the lower quadrant of the left breast (Fig. 5). There was no thickening and/or increase in the surrounding breast trabeculae, and no swelling in the axillary lymph nodes. The other 4 cases were located under the left axilla, and in the left thigh muscles (Fig. 6), the left lobe of the liver (Fig. 7), and the right partes iliaca region (Fig. 8), respectively. Compared with surrounding muscle tissues, these lesions were characterized by slightly irregular higher- or lower-density masses with unclear boundaries with adjacent tissues. Besides, 2 of these lesions presented massive calcification and ossification (Figs. 6 and 8). CT scan were performed on these 4 cases with CT number ranging from 28 to 61 HU. The contrast-enhanced CT images (CT number change: < 10 HU) of 2 participants revealed no obvious enhancement, while significant enhancement was observed in another case.

### MRI findings

**Fig. 9:**
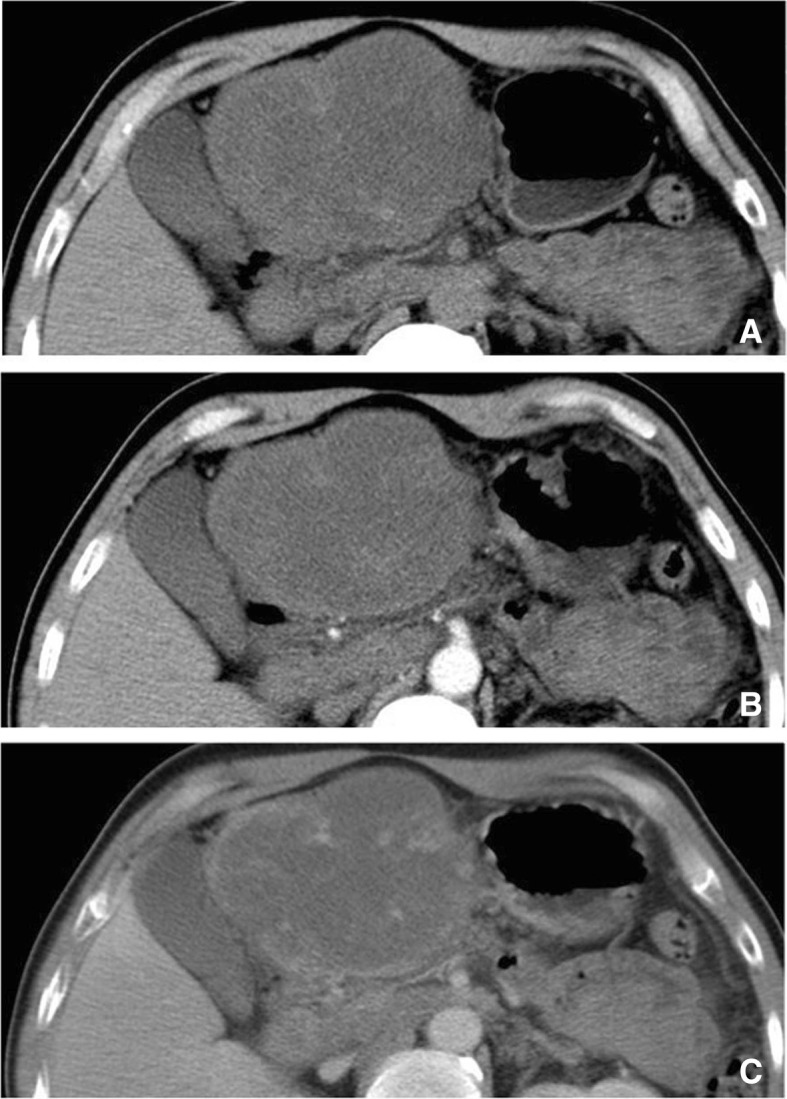
LGMS of the right partes iliaca. X-ray revealed an irregular area of ossification above the right greater trochanter of the femur

MRI results are illustrated in Table 1. Routine preoperational contrast-enhanced MRI was performed on 5 cases. As far as T1-weighted images (T1WI) were concerned, all of the 5 cases presented homogeneous iso- or hyper-signal intensities. Short T1 and long T2 signals (indicating hemorrhage) were observed in one participant. As for the T2-weighted images (T2WI), all of the 5 tumors presented either homogeneous or heterogeneous hyper-signal intensities (Fig. 9b and 3b). No obvious necrosis or cystic components were observed. Contrast-enhanced MR images revealed homogeneous signal enhancement (2 cases, Fig. 9c), heterogeneous enhancement (1 case) and rim enhancement (2 cases, Fig. 3c).

### Histopathological findings

During surgical procedures, tumors were taupe and pale yellow in color and were partially circumscribed or non-encapsulated. They adhered to the surrounding tissues and invaded muscular structures and adipose tissues. Large amounts of blood vessels were seen in some tumors with a rotten-fish-like look. Microscopic examinations of the tumor cells revealed diffuse infiltrative growth without distinguishable margins with surrounding tissues. Some tumor cells were bulky, fusiform or irregularly shaped, undergoing occasional mitosis and mild interstitial myxoid change and accompanied by a small amount of inflammatory cell infiltration. Tumor necrosis was present with local hyaline degeneration. Four cases presented calcium deposits and bone metaplasia. Immunohistochemistry analysis results were Vim (+), CD99 (+), desmin (+), NSE (some cells +), CD117 (−), SMA (+), S-100 (−), and ki-67 (1–60%).

## Discussion

Myofibroblastic tumor cell is a type of contractible fusiform mesenchymal cell. It integrates the morphologic features of fibroblasts and those of smooth muscle cells into itself [2]. It was firstly discovered in granulation tissues. Mentzel et al. [2] reported 18 cases of low-grade myofibroblastic sarcoma in 1998. This type of tumor was recognized as a new category by the World Health Organization’s (WHO) in *“Pathology and Genetics of Tumors of Soft Tissue and Bone”* in 2002, and characterized by intermediate grade (occasionally transferable) malignancy. Tumor cells were considered as the origin of myofibroblasts. The WHO 2013 document maintained the classification of this disease [3]. Accurate categorization of myofibroblastic tumors and inflammatory myofibroblastic tumors (IMT) on oncology may provide further insights into pseudo-tumoral lesions and myofibroblastic tumors.

To the best of our knowledge, only a few studies about LGMS have been conducted, reporting 65 cases of this disease [4–9]. Tumors occurred to 24 cases in the head and neck region (24/65, 38.7%), including the lower jaw, jawbone, nasal sinus, oral cavity, etc. Among the other 41 cases, tumors were found in the upper and lower limbs, ileums, bones, etc. In these patients with an average age of 40 years old (age range: 9–75 years), most of the tumors were solitary with a maximum diameter of 1.5 to 22 cm. Only 3 cases of them were reported to develop tumors in the abdomen, pelvis and upper extremities [2, 10]. All of our participants (average age: 45.5 years old) developed solitary tumors, with the largest maximum diameter being 13.5 cm. Five out of these tumors were located in bones (35.7%), and most of the 5 were in the distal femur. Another five participants’ tumors were located in the musculoskeletal groups of the extremities (35.7%). The other four cases developed tumors in the pyriform sinus, epiglottis, breast and liver (7.1%), respectively. Overall, the tumor locations in our studies were different from those reported previously.

Although LGMS is classified as an intermediate grade tumor, high recurrence and metastasis rate highlight the need for more pathological analyses. Among the 65 cases mentioned above [5–9] and the 14 cases in our study, overall 23 cases suffered local recurrence (23/79, 29.1%), among which 3 were from our study (3/14, 21.4%). The recurrence period varied from 6 months to 7 years, with a maximal recurrence frequency of 4 times. After 3 recurrences of inflammatory myofibroblastic tumor, one participant in our study (Case 14) developed LGMS and the following 4 recurrences were all LGMS, indicating an increase in tumor grade due to recurrence. Theoretically, recurrence of LGMS can lead to escalation to intermediate-grade and high-grade myofibroblastic sarcoma. However, to the best of our knowledge, no such escalation has yet been reported. Fourteen cases experienced distal metastases (14/79, 17.7%), among which 5 were from our study (5/14, 35.7%). This means that the overall metastasis rate among our 14 participants is obviously higher than that among the 65 cases previously reported. Metastasis and the primary tumor were found at the same time in only 1 case (Case 1), while in the other 13 cases of our study, metastases were found within 4 months to 9 years after surgical procedures. The most common sites of metastasis include the lungs (7/14) and bones (5/14). Metastasis was also found in the heart, gastrocnemius, and tongue. Multiple metastases may occur to any of these body parts. There was one patient who underwent extensive resection and radiotherapy, and did not experience any recurrence or metastasis within 12-year follow-up. Other 6 patients who underwent extensive resection suffered no recurrence within 18 to 59 months of the follow-up. Our findings demonstrate that extensive resection of LGMS contributes to a good prognosis. Only a few tumors (especially those in deep locations) recur after local excision (mostly within 2 years, including small tumors), and some of these tumors may metastasize to distal locations 5 years later. After a series of evaluation and grading, we come to a conclusion: patients suffering metastasis but expecting to live longer can undergo extensive resection, joint replacement and adjuvant chemotherapy or radiotherapy, while palliative therapy can be prescribed to those who are intolerable to these therapies. So far, 2 patients have died of multiple organ failures. Previous studies [10] have reported that intermediate grade myofibroblastic sarcomas have a high rate of recurrence and metastasis, compared with fibrosarcoma and leiomyosarcoma.

Scarcity of imaging studies of LGMS makes imaging characteristics of this disease still poorly understood. To our knowledge, imaging findings about 4 LGMS cases have been reported so far [5, 7–9], and the tumors were found in the wall of the right atrium, abdomen, epiglottis, and distal femur, respectively. The tumor located in the right atrium was a result of metastasis. It was more hyperintense than that in the myocardium on T1WI images, and the signal was enhanced on delayed contrast-enhanced images [8]. Another case of a large abdominal LGMS was characterized by a low signal solid mass with significant signal enhancement in the early phase and concentric filling during the late phase. MRI of this tumor located in abdomen displayed homogeneous and hypointense signal on T1WI, hyperintense signal on T2WI, and homogeneous signal enhancement on contrast-enhanced images. Enhanced CT scan of the tumor located in the epiglottis revealed inhomogeneous enhancement [5]. The patient whose tumor was located in the distal femur presented extensive multi-cystic bone destruction with clearly sharp margins and without significant hardened edges. Parts of the cortical bone were invaded and a soft tissue mass developed. In all four cases described above, no calcification or ossification of the lesions was observed, and there was no CT number change before and after enhanced CT scans. Some tumors in our study were located in bones (5/14,35.7%) with 3 of the 5 in the distal femur (3/5, 60%). Imaging results in our study are different from those previously reported [9]. The 3 tumors located in the femur were in high-grade malignancy, and with permeant tumor tissues and osteolytic bone destruction. In addition, the bone interval boundary was not clear, and transitional zone and cortical bone erosion were present. In the other 2 cases whose tumor were located in the right shoulder blade and right lower pubic region, the huge mass damaged the bone cortex and a significant soft tissue mass formed. Massive ossification was observed in the primary tumor and the lung metastasis (Case 1). X-ray or CT scan of 2 LGMS cases revealed significant ossification masses within bones, indicating a higher rate of LGMS ossification than that reported in previous studies. LGMS ossification is presumably attributed to multiple differentiations of intratumoral myofibroblasts into metaplasia-induced osteoblasts. There have been no reports about osteoblastic metastases from LGMS. It is important to distinguish bone LGMS from osteosarcoma, fibrosarcoma, malignant fibrous histiocytoma or malignant cartilaginous tumor.

Breast LGMS is very rare. To our knowledge, 1 of our 14 cases represents the fourth reported case [11–13]. It is necessary to differentiate breast LGMS from breast cancer. Most breast sarcomas of mesenchymal origins are prone to blood metastasis, while breast cancer of epithelial origins are prone to lymphatic metastasis. Therefore, mammography of advanced-stage breast cancers usually reveals interstitial edema, increased trabecular thickening, opacity of the subcutaneous fat layer and thickening of the skin because of the clogged lymphatic drainage from the cancer cells. However, these and axillary lymph node enlargement are not observed in the mammographs of stromal breast sarcoma. Breast LGMS in our study is characterized by a certain level of aggressiveness (indicated by an irregular shape, and multiple angular and poorly-circumscribed margins), or precisely, a lack of encapsulation.

## Conclusions

LGMS is a rare type of tumor. Only few systematic studies of its diagnostic and radiological features have been conducted. In this study, we find that LGMS is characterized by several imaging features: invasiveness, metastasis and calcification. Inflammatory myofibroblastic tumors are likely to progress into LGMS after several recurrences. A large portion of the tumors in our study were located in bones. This study has two limitations. Only small amounts of patients were recruited. And more imaging examinations should have been performed on each patient. Therefore, further exploration is need to understand imaging features of LGMS better, for example, the most preferred site. Although it is classified as an intermediate grade tumor, LGMS has high rate of recurrence, metastasis and calcification, which also highlights the need for further clinical and pathological analysis. With accurate diagnosis comes effective treatment.

## Abbreviations

CT: Computer tomography
LGMS: Low-grade myofibroblastic sarcoma
MRI: Magnetic resonance imaging
